# Metabolic Obesity Phenotypes and Risk of Lung Cancer: A Prospective Cohort Study of 450,482 UK Biobank Participants

**DOI:** 10.3390/nu14163370

**Published:** 2022-08-17

**Authors:** Fang Shao, Yina Chen, Hongyang Xu, Xin Chen, Jiawei Zhou, Yaqian Wu, Yingdan Tang, Zhongtian Wang, Ruyang Zhang, Theis Lange, Hongxia Ma, Zhibin Hu, Hongbing Shen, David C. Christiani, Feng Chen, Yang Zhao, Dongfang You

**Affiliations:** 1 Department of Biostatistics, School of Public Health, Nanjing Medical University, Nanjing 211166, China; 2 Department of Critical Care Medicine, Wuxi People’s Hospital Affiliated to Nanjing Medical University, Wuxi 214023, China; 3 Department of Environmental Health, Harvard T.H. Chan School of Public Health, Boston, MA 02115, USA; 4 China International Cooperation Center of Environment and Human Health, Nanjing Medical University, Nanjing 211166, China; 5 The Center of Biomedical Big Data and the Laboratory of Biomedical Big Data, Nanjing Medical University, Nanjing 211166, China; 6 Section of Biostatistics, Department of Public Health, Faculty of Health and Medical Sciences, University of Copenhagen, ØsterFarimagsgade 5, 1353 Copenhagen, Denmark; 7 Department of Epidemiology, School of Public Health, Nanjing Medical University, Nanjing 211166, China; 8 Jiangsu Key Lab of Cancer Biomarkers, Prevention and Treatment, Collaborative Innovation Center for Cancer Personalized Medicine, Nanjing Medical University, Nanjing 211166, China; 9 Department of Medicine, Massachusetts General Hospital/Harvard Medical School, Boston, MA 02115, USA

**Keywords:** metabolically healthy obesity, lung cancer, metabolic obesity phenotypes, Mendelian randomization

## Abstract

(1) Background: The association between metabolic obesity phenotypes and incident lung cancer (LC) remains unclear. (2) Methods: Based on the combination of baseline BMI categories and metabolic health status, participants were categorized into eight groups: metabolically healthy underweight (MHUW), metabolically unhealthy underweight (MUUW), metabolically healthy normal (MHN), metabolically unhealthy normal (MUN), metabolically healthy overweight (MHOW), metabolically unhealthy overweight (MUOW), metabolically healthy obesity (MHO), and metabolically unhealthy obesity (MUO). The Cox proportional hazards model and Mendelian randomization (MR) were applied to assess the association between metabolic obesity phenotypes with LC risk. (3) Results: During a median follow-up of 9.1 years, 3654 incident LC patients were confirmed among 450,482 individuals. Compared with participants with MHN, those with MUUW had higher rates of incident LC (hazard ratio (HR) = 3.24, 95% confidence interval (CI) = 1.33–7.87, *p* = 0.009). MHO and MHOW individuals had a 24% and 18% lower risk of developing LC, respectively (MHO: HR = 0.76, 95% CI = 0.61–0.95, *p* = 0.02; MHO: HR = 0.82, 95% CI = 0.70–0.96, *p* = 0.02). No genetic association of metabolic obesity phenotypes and LC risk was observed in MR analysis. (4) Conclusions: In this prospective cohort study, individuals with MHOW and MHO phenotypes were at a lower risk and MUUW were at a higher risk of LC. However, MR failed to reveal any evidence that metabolic obesity phenotypes would be associated with a higher risk of LC.

## 1. Introduction

Lung cancer (LC) continues to be the leading cause of cancer morbidity and mortality worldwide over the last several decades [1]. More than 2.2 million new LC cases and nearly 1.8 million deaths were estimated in 2020, accounting for 11.4% of the global cancer burden and 18.0% of all cancer deaths [2]. Known risk factors of LC typically include cigarette smoking, age, gender, family history, and occupational exposure [3,4].

Overweight/obesity has also become an irresistible global epidemic over the past 50 years, with over 2 billion people, about 30% of the world population [5,6]. Generally, obesity can lead to metabolic abnormalities such as insulin resistance, increased blood pressure (BP), prediabetes, dyslipidemia, and metabolic syndrome [7,8]. However, people with obesity have variability in metabolic factors. It has been reported that a subset of individuals with obesity do not develop metabolic disorders, and they are described as having a metabolically healthy obesity (MHO) phenotype [9]. More notably, the prevalence of metabolic obesity phenotypes varies between 10% and 30% [10,11,12], and prevalence has been found higher in women than men and seems to decrease with age in both genders [13].

Although obesity is typically associated with a higher risk of most cancer types, several recent systematic reviews have demonstrated an “obesity paradox” in which obesity has a potentially protective effect on LC risk [14,15]. Metabolic status such as insulin resistance [15], high BP [16], and low-density lipoprotein cholesterol (LDL-C) [17] have been proven to be positively associated with an increased risk of LC. However, it remains unclear how metabolic status differs among different BMI groups and whether metabolic obesity phenotypes affect LC risk.

Therefore, we examine the association of eight metabolic obesity phenotypes, which are defined at baseline by combinations of body weight and metabolic health status, with the risk of LC in 450,482 participants in UK Biobank.

## 2. Materials and Methods

### 2.1. Study Population

A total of 502,461 individuals without withdrawals aged 40–69 years at baseline (94% of self-reported European ancestry) living within 25 miles of one of the 22 study assessment centers across the UK were recruited into UK Biobank from 2006 to 2010 in this prospective cohort study (Project ID: 52785). Counts of participants in this study were updated August 2021. A comprehensive set of individual-level data was provided by participants through touch-screen questionnaires, face-to-face interviews, physical examinations, and provided biological samples [18]. In this study, we excluded participants who were diagnosed with malignant neoplasms (excluding other malignant neoplasms of skin) at baseline (*n* = 51,928) or lung cancer within a year (n=51). Finally, a total of 450,482 participants were included in the analysis (Supplement Figure S1).

### 2.2. Measurement of Adiposity and Metabolic Factors

Height was measured to the nearest centimeter using a Seca 202 stadiometer (Hamburg, Germany), and bodyweight was measured to the nearest 0.1 kg using a Tanita BC-418 body composition analyzer (Tokyo, Japan) during the initial assessment center visit [19]. BMI was calculated as weight in kilograms divided by height in meters squared. UK Biobank collects about 45 mL of blood from each participant using the vacutainer system at the initial assessment center visit [20]. Blood samples from participants are transported overnight by commercial courier to a central laboratory where they are processed and analyzed by a Beckman automated hematology analyzer (Beckman Coulter AU5800). High-sensitivity CRP (hs-CRP), HDL-C, LDL-C, and triacylglycerols were measured by immuno-turbidimetric, enzyme immuno-inhibition, enzymatic selective protection, and enzymatic automatically. Each process step included quality assurance procedures, and the data outputs have an associated component that identifies the machine and operator involved in processing the sample [21]. The HbA1c assay was performed using five Bio-Rad Variant II Turbo analyzers by Bio-Rad Laboratories. BP was measured by registered nurses using an Omron 705 IT electronic blood pressure monitor (or manually if unavailable), and the mean of available measurements was derived [21].

### 2.3. Assessment of Metabolic Obesity Phenotypes

BMI categories were defined according to the World Health Organization 2000 criteria [22]: underweight (BMI <18.5 kg/m${}^{2}$), normal weight (BMI 18.5–25 kg/m${}^{2}$), overweight (BMI 25–30 kg/m${}^{2}$), and obese (BMI ≥30 kg/m${}^{2}$). We defined metabolic health based on the original NCEP ATP III definition [23] and previous studies [24]: (i) systolic/diastolic blood pressure <130/85 mmHg; (ii) C-reactive protein <3 mg/L; (iii) triacylglycerols <2.3 mmol/L; (iv) LDL-C <3 mmol/L and no cholesterol-lowering medications; (v) HDL-C >1 mmol/L; (vi) HbA1c <42 mmol/mol and no diabetes medications (Supplement Table S1). At baseline, participants who met 4 of the 6 criteria above were considered metabolically healthy. Based on the combination of BMI categories and metabolic health status, participants were then categorized into eight groups: metabolically healthy underweight (MHUW), metabolically healthy normal (MHN), metabolically healthy overweight (MHOW), metabolically healthy obesity (MHO), metabolically unhealthy underweight (MUUW), metabolically unhealthy normal (MUN), metabolically unhealthy overweight (MUOW) and metabolically unhealthy obesity (MUO). In order to explore the trend association of groupings, we define two orders, one is MHUW, MUUW, MHN, MUN, MHOW, MUOW, MHO, MUO (trend1) [25], and the other is MHUW, MHN, MHOW, MHO, MUUW, MUN, MUOW, MUO (trend2) [26].

### 2.4. Ascertainment of LC

Incident lung cancer cases were ascertained through cancer registry records using the 10th Revision of the International Classification of Diseases (ICD)-10 coding and coded as C33-C34 [27,28]. The lung cancer diagnoses of the residents were identified by linking data from hospital episodes statistics (HES) data and the National Cancer Registry. Participants free of lung cancer were followed up to the date of diagnosis, loss to follow-up, death, or date of complete follow-up, whichever occurred first [29]. The censoring date varies between cancer registries, and the cancer register data of Scotland and England and Wales are updated to 31 October 2015 and 31 July 2019 as the complete follow-up, respectively. The hospital admission data were available up to 31 March 2021 for participants in England and Scotland, and 28 February 2018 for those in Wales.

### 2.5. Covariates

Sociodemographic and behavioral risk factors were included as covariates, as they could potentially confound the association between metabolic obesity phenotypes and lung cancer [28]. Age was calculated using birth date and the date of baseline assessment. Sex of participant was acquired from the central registry at recruitment and updated by the participant. Education was based on self-report of the highest qualification achieved and dichotomized into university/college degree or less [30,31]. Ethnicity was self-reported and categorized into: White, South Asian, Black, Chinese, other, and mixed [32]. The smoking status variable divided participants into never smokers and former or current smokers. Duration of smoking was calculated using the age last smoked cigarettes regularly minus the age when started smoking regularly [33]. Family history of lung cancer was derived from illness history of first-degree relatives, and personal history of emphysema/bronchitis was self-reported.

### 2.6. Genotyping

Genotyping of UK Biobank participants was performed with one of two arrays (The Applied Biosystems UK BiLEVE Axiom Array (Affymetrix) and Applied Biosystems UK Biobank Axiom Array). Sample quality control (QC) measures included removing individuals who were duplicated, had sex mismatches, or those identified to be outliers of heterozygosity (±6SD) and of a high missing rate (≥0.05). Criteria at the single nucleotide polymorphism (SNP) level were mapped to autosomal chromosomes, minor allele frequency (MAF) ≥0.01, *p*-value for Hardy–Weinberg equilibrium (HWE) among LC individuals $\geq 1.0\times 10^{-12}$, and genotype missing rate ≤0.05. After quality control analysis, a total of 482,958 unduplicated individuals with 94,796 SNPs were used for analysis. Further details of the QC measures applied and imputation performed have been described previously [34,35].

### 2.7. Statistical Analyses

Normally distributed continuous variables are described as means (SDs) and compared using unpaired, 2-tailed *t*-tests. Categorical variables were compared using $\chi^{2}$ tests. The multivariate Cox proportional hazard regression models were used to estimate the hazard ratios (HRs) and their 95% confidence intervals (CIs) between metabolic obesity phenotypes, BMI groups, metabolic health status and LC risk with adjustment for age, sex, education level, ethnicity, smoking status, smoking duration, family history of LC and personal history of emphysema/bronchitis.

We also performed stratified analysis to evaluate the heterogeneity of the association between metabolic obesity phenotypes, BMI groups, metabolic health status and LC risk stratified by smoking status, gender or age. Furthermore, we performed a series of sensitivity analyses to assess the reliability of association results. Model 1 did not exclude the individuals who were diagnosed with LC in the first year of follow-up; Model 2 was additionally adjusted for family history of any cancer and drinking status; Model 3 was additionally adjusted for age at first smoking, the number of cigarettes smoked daily, package year, and time since last smoking (in former smokers); Model 4 excluded former smokers; Model 5 excluded participants that were non-Europeans; Model 6 excluded participants with missing covariates; Model 7 additionally made an adjustment for dietary patterns (red and processed meat intake, fruit and vegetable intake) and physical activity (IPAQ); Model 8 additionally made an adjustment for waist–hip ratio (as a continuous variable).

In one-sample Mendelian randomization (MR) analysis, we used instrumental variable analysis by two-stage least-squares regression (2SLS) to estimate the potential causal association between individuals with metabolic obesity phenotypes and risk of LC. For subgroups of MHO, BMI, and metabolic groupings, we used the factorial MR as previously reported [36]. In 2SLS, the exposure of interest is regressed on the polygenic score, and the outcome is regressed on the predicted values of the exposure from the first regression. All 2SLS models were adjusted for age, sex, the top 10 principal components, and the specific genotyping array used. For each biomarker, we constructed a weighted allele score based on SNPs that passed a *p*-value threshold *p* $<5\times 10^{-8}$ and pruned based on the European-population LD reference panel with an $r^{2}<0.001$ and a 1 MB clumping window to obtain independent genetic predictors. We relaxed the instrument *p*-value threshold ($p<5\times 10^{-6}$) for several traits lacking sufficient SNPs (≤3) after LD. The Cragg–Donald *F*-statistic was used to estimate the strength of the association, and *F* values >10 were regarded as useful for MR analysis [37,38].

A two-sample MR analysis was performed using the “TwoSampleMR” R package. Consistent with one-sample MR analysis, the same threshold (LD $r^{2}<0.001$ and $p<5\;\times \;10^{-8}$) was used to include more genetic variants and maximize the strength of instruments in the regression of metabolic obesity phenotypes, BMI groups and metabolic health status upon individual SNPs. Summary statistics between LC and LC SNPs in Europeans were taken from the International Lung Cancer Consortium (ILCCO) and derived from the “MR-base” R package with 11,348 European cases [39]. We used the inverse variance-weighted (IVW) meta-analyses as the primary method, which uses weighted linear regression and is equivalent to two-stage least squares or allele score analysis using individual-level data. We also performed sensitivity analyses to assess for pleiotropy using simple median, weighted median, and MR-Egger [40].

Furthermore, we detected heterogeneity in Cox regression stratified by smoking status, so we applied a multi-stratum Mendelian randomization analysis. We stratified individuals on smoking status (never smokers, *n* = 246,380; former smokers, *n* =152,785; current smokers, *n* = 48,660, Supplement Table S2). Then, genome-wide association studies and one-sample Mendelian randomization analysis were performed for metabolic obesity phenotypes, BMI groups, metabolic health status and LC in UK Biobank adjusted for age, sex, the top 10 principal components and the specific genotyping array used, restricted to only the individuals in each respective subset. All *p*-values were two-sided, and p<0.05 was considered statistically significant. Statistical analyses were performed using R software (version 4.1.2, R Foundation for Statistical Computing, Vienna, Austria).

## 3. Results

### 3.1. Participant Characteristics

During a median follow-up of 9.1 years (IQR 6.6–11.6 years), 3654 incident LC patients were confirmed. Table 1 shows the baseline characteristics of the participants. Among the 450,482 participants, the mean (SD) age at baseline was 56 (8) years, and 45.5% were men. At baseline, 28.5% (*n* = 128,167) of the participants were metabolically unhealthy, and 24.5% (*n* = 110,408) had obesity. The MHO phenotype accounted for 7.6% (*n* = 34,117) of the total population and 30.9% of the obese population. The prevalence of metabolic unhealthy phenotypes was higher in LC people than in non-LC people at baseline. Metabolically healthy individuals were more likely to be younger and female across all BMI categories (Supplement Figure S2).

### 3.2. Association of Metabolic Obesity Phenotypes with LC Risk

**Figure 1:**
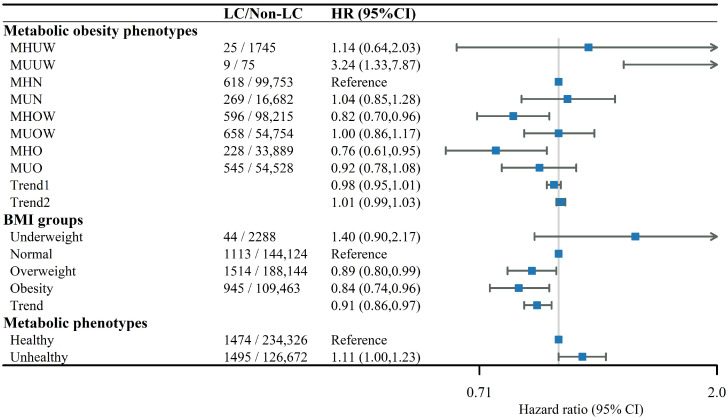
Association between metabolic obesity phenotypes and LC. HRs: hazard ratios; LC: lung cancer; BMI, body mass index; MHUW, metabolically healthy underweight; MHN, metabolically healthy normal; MHO, metabolically healthy obesity; MHOW, metabolically healthy overweight; MUUW, metabolically unhealthy underweight; MUN, metabolically unhealthy normal; MUO, metabolically unhealthy obesity; MUOW, metabolically unhealthy overweight.

Compared with participants with MHN at baseline, those with MUUW had higher rates of incident LC (hazard ratios (HR) = 3.24, 95% confidence interval (CI ) = 1.33–7.87, *p* = 0.009, Figure 1). However, the limited LC cases means that we cannot absolutely assume that individuals with the MUUW phenotype are at higher risk for LC. MHO individuals had a 24% lower risk of developing LC (HR = 0.76, 95% CI = 0.61–0.95, *p* = 0.02) compared with MHN individuals, and the corresponding HR for MHOW was 0.82 (HR = 0.82, 95% CI = 0.70–0.96, *p* = 0.02). For BMI groups, the HRs for the overweight and obesity individuals were 0.89 (0.80–0.99, p=0.04) and 0.84 (0.74–0.96, p=0.009). In addition, we found a lower risk of LC with the increase of BMI groupings. However, metabolic unhealthy individuals had a higher risk of LC (HR = 1.11, 95% CI = 1.00–1.23, p=0.04). Furthermore, the mutually adjusted associations of BMI groups and the metabolic health groups with LC remained consistent (Supplement Table S3).

The smoking-, sex-, and 60 years age-stratified association analyses are presented in Supplement Figures S3–S5. For former smokers, the HRs for the MUUW, MUOW and MUO individuals were 24.28 (6.00–98.31, *p* = 7.8 $\times \;10^{-6}$), 1.59 (1.28–1.99, *p* = 4.0 $\times \;10^{-5}$) and 1.61 (1.30–2.01, *p* = 2.0 $\times \;10^{-5}$) for LC, respectively. The risk of lung cancer increased significantly from MHUW to MUO for both orders ($P_{trend1}=6.7\;\times \;10^{-5},P_{trend2}=1.3\;\times \;10^{-7}$). However, for current smokers, MHOW individuals had a 39% lower risk of developing LC (HR = 0.61, 95% CI = 0.49–0.77, *p* = 3.0 $\times \;10^{-5}$) compared with MHN individuals. Notably, stratified analyses by smoking status showed that there is heterogeneity in the effect of smoke status-specific MHO, BMI and metabolic phenotypes on LC risk (not all *p*-values are greater than 0.05, Supplement Figure S3). In the male subgroup, the HRs for the MUUW individuals were 5.59 (2.06–15.2, *p* = 0.001), whereas MHOW and MUO individuals had a 24% and 23% lower risk of developing LC (MHOW: HR = 0.76, 95% CI = 0.60–0.97, *p* = 0.02; MUO: HR = 0.77, 95% CI = 0.60–0.98, *p* = 0.03) compared with MHN male individuals. The risk of lung cancer decreased significantly from MHUW to MUO for both orders ($P_{trend1}=0.01,P_{trend2}=0.01$). MUUW individuals less than 60 years old had the higher risk of developing LC (HR = 12.10, 95% CI = 3.81–38.5, *p* = 2.4 $\times \;10^{-5}$).

The sensitivity analyses detailed in methods showed that the primary model retained a stable association between MHO individuals and LC risk in Model 1, Model 2, Model 3, Model 6, Model 7 and Model 8 (Supplement Table S4). The protective effect of MHOW on lung cancer remained stable in Model 1, Model 4, Model 5, Model 6, Model 7 and Model 8. MUUW individuals’ associations did not change in Model 1, Model 3, Model 5, Model 6 and Model 8. Furthermore, the association of individuals with BMI groups and LC were not materially altered in all sensitivity analyses, and the association of individuals with metabolic health status and LC were stable in Model 1, Model 5 and Model 6.

### 3.3. MR Analysis of Metabolic Obesity Phenotypes and LC Risk

The number of SNPs included in each polygenic score, *F*-statistics and the corresponding effect estimates from the 2SLS regression are shown in Table 2. *F*-statistics for the polygenic scores ranged from 31 to 657, which suggests that they were not weak instruments. We detected no evidence for a significant causal effect of metabolic obesity phenotypes, BMI groups, or metabolic phenotypes on the LC risk, with close-to-zero effect estimates (all *p*-values >0.05).

In contrast, we found statistically significant evidence of a relationship between individuals with MUUW, MHO, MUO and LC in two-sample MR (MUUW:OR = 0.98, 95% CI = 0.97–1.00, *p* = 0.03; MHO: OR = 1.07, 95% CI = 1.01–1.14, *p* = 0.03; MUO:OR = 1.11, 95% CI = 1.05–1.17, *p* = 0.001, Table 3). The risk of lung cancer increased significantly from MHUW to MUO for MHUW/MUUW/MHN/MUN/MHOW/MUOW/MHO/MUO order (trend1MHO:OR = 1.17, 95% CI = 1.07–1.27, $P_{trend1}=2.9\;\times \;10^{-4}$). Consistent results across BMI groups (obesity: OR = 1.09, 95% CI = 1.04–1.14, *p* = 0.001; trendBMI: OR = 1.35, 95% CI = 1.14–1.60, $P_{trend1}=4.9\;\times \;10^{-4}$). The results of the additional sensitivity analysis are presented in the Supplement Table S5.

In multi-stratum Mendelian randomization analyses, we observed a significant decrease in the risk for lung cancer across the groups of the MHO trend2 and BMI trend in current smokers (trend2MHO:OR = 0.99, 95% CI = 0.98–1.00, $P_{trend1}=0.04$; trendBMI: OR = 0.97, 95% CI = 0.95–0.99, $P_{trend}=0.007$). No meaningful differences were observed in never smokers or former smokers (Supplement Tables S6–S8).

## 4. Discussion

In this large, prospective, population-based cohort study, we observed that individuals with MHOW and MHO phenotypes were at lower risk for lung cancer, and individuals with MUUW were at a statistically significantly higher risk of LC. However, the Mendelian randomization analyses failed to provide genetic evidence that metabolic obesity phenotypes were associated with incident LC.

To the best of our knowledge, this was the first study to systematically investigate the association between metabolic obesity phenotypes and incident LC. Our study added to emerging evidence that metabolic obesity phenotypes may be observationally associated with the risk of LC. However, a previous observational study [41] involving site-specific cancers of UK Biobank failed to find a significant association between the two. Different inclusion and exclusion criteria, different metabolic health criteria and different covariates may account for the differences in results. In addition, previous studies have shown that being underweight is associated with higher incidence and poor survival [42], which is similar to our MUUW results. Furthermore, our findings are consistent with previous cohort studies that have reported an inverse association between BMI and LC risk, and being overweight or obese was associated with a lower risk of LC [42,43]. Similarly, several previous cohorts observed an inverse association between BMI and risk of LC among current smokers rather than former and current smokers [44,45]. However, we did not find a protective effect of being overweight or obese in former smokers; instead, our observational results showed a higher risk effect between the two, contrary to previous studies [46]. Differences by sex have been assessed in previous studies that BMI was statistically significantly inversely associated with the risk of lung cancer only among male rather than female [47], which is similar to our findings.

Mendelian randomization evaluating the association between genetic polymorphisms that affect metabolic obesity phenotypes and incident LC revealed neutral, nonsignificant associations, again supporting the absence of an association between metabolic obesity phenotypes and LC risk. In contrast to our observational analysis, we detected no evidence for a significant causal effect of metabolic obesity phenotypes, BMI groups, or metabolic phenotypes on the LC risk. Although it may be a true phenomenon, the results of Mendelian randomization studies warrant a cautious interpretation. Mendelian randomization is considered a powerful tool to infer causality from nature’s randomization, but it is not completely protected from bias and confounders [48]. For example, metabolic obesity phenotypes at baseline were time-dependent, which depended on the influence of the acquired environment. Although genetic variants contributed to metabolic obesity phenotypes, it is possible that the attenuation or reverse of an effect on LC may have been caused by the time-dependent exposure [49]. In addition, the polygenic score explains only a relatively small proportion of the variation in metabolic obesity phenotypes. Summary-level genetic association statistics in two-sample MR may be derived from different study designs such as case-control, case-only and cohort studies, which further inevitably affects the causal effect estimation of exposure on the outcome or even leads to opposite results [50]. In the end, however, this study found no consistent evidence from these genetic analyses that a greater propensity to metabolic obesity phenotypes increased the risk of LC.

Our study has multiple strengths, including a community-based prospective cohort design with a substantial sample size to investigate the association between metabolic obesity phenotypes and LC, careful control for established and potential risk factors, and the measurement of six metabolic markers using a validated technique. Additionally, serum samples were collected before the diagnosis of LC. Hence, the possibility of reverse causality could be ruled out. Furthermore, there was no systematic study between metabolic obesity phenotypes and LC risk before; this was the first study to systematically investigate the association between the two.

As for limitations, this study did not consider alternative definitions of MHO [28,51]. For example, some studies [52,53] defined MHO as obesity in the absence of metabolic diseases such as hypertension, dyslipidemia, and type 2 diabetes. For others [54], the definition was to meet almost all the metabolic criteria. Therefore, the prevalence of MHO varied depending on the definitions used [52,55]. Furthermore, the limited LC cases with MUUW showed the decreased statistical power of our analysis. In addition, as with many observational studies, although exhaustive adjustment was performed in the multivariable analyses, residuals or unmeasured confounding factors cannot be excluded [56,57]. Metabolic obesity phenotypes measured neither body composition nor fat distribution [58], which would be associated with increased LC risk. In addition, metabolic health status changed over time, which meant the physiological state at baseline had a great influence on the metabolic markers’ concentration. Finally, some previous studies demonstrated that BMI and metabolic status have different or even opposite effects on different pathological types of LC [59]. Future studies may consider separate associations with different pathological types of LC as outcomes.

As a newly defined disease, metabolic obesity phenotypes contained information on both BMI and metabolic status and should receive more attention. In future clinical practice, on the one hand, BMI and metabolic intervention in high-risk populations should be enhanced to prevent LC. Then, individual treatment decisions based on the metabolic obesity phenotypes should be developed. On the other hand, on the research side, potentially genetic instruments for metabolic obesity phenotypes should be explored, and the definition of metabolic health needs to be harmonized [60]. Furthermore, future studies could consider taking advantage of metabolic obesity phenotypes as a model to understand the potential mechanisms of BMI, fat distribution [22], long-term diet pattern [58], and dysfunction of LC [61].

## 5. Conclusions

In this prospective cohort study, individuals with MHOW and MHO phenotypes were at a lower risk and individuals with MUUW were at a higher risk of LC compared with individuals with MHN phenotypes. However, Mendelian randomization failed to reveal any evidence that metabolic obesity phenotypes would be associated with a higher risk of LC. Our results provided additional evidence for the role of metabolic obesity phenotypes in lung cancer risk.

## Figures and Tables

**Table 1:** Baseline characteristics of lung cancer cases and controls in the UK Biobank.

Characteristics	Total (*n* = 450,482)	LC (*n* = 3654)	Non-LC (*n* = 446,828)	*p*-Value
Metabolic obesity phenotypes, N (%)				<0.001
MHUW	1770 (0.4)	25 (0.7)	1745 (0.4)	
MUUW	84 (0.0)	9 (0.2)	75 (0.0)	
MHN	100,371 (22.3)	618 (16.9)	99,753 (22.3)	
MUN	16,951 (3.8)	269 (7.4)	16,682 (3.7)	
MHOW	98,811 (21.9)	596 (16.3)	98,215 (22.0)	
MUOW	55,412 (12.3)	658 (18.0)	54,754 (12.3)	
MHO	34,117 (7.6)	228 (6.2)	33,889 (7.6)	
MUO	55,073 (12.2)	545 (14.9)	54,528 (12.2)	
Missing	87,893 (19.5)	706 (19.3)	87,187 (19.5)	
BMI groups, N (%)				<0.001
Underweight	2332 (0.5)	44 (1.2)	2288 (0.5)	
Normal	145,237 (32.2)	1113 (30.5)	144,124 (32.3)	
Overweight	189,658 (42.1)	1514 (41.4)	188,144 (42.1)	
Obesity	110,408 (24.5)	945 (25.9)	109,463 (24.5)	
Missing	2847 (0.6)	38 (1.0)	2809 (0.6)	
Metabolic phenotypes, N (%)				<0.001
Healthy	235,800 (52.3)	1474 (40.3)	234,326 (52.4)	
Unhealthy	128,167 (28.5)	1495 (40.9)	126,672 (28.3)	
Missing	86,515 (19.2)	685 (18.7)	85,830 (19.2)	
Age, mean ± SD	56 ± 8	61 ± 6	56 ± 8	<0.001
Sex, N (%)				<0.001
Female	245,422 (54.5)	1840 (50.4)	243,582 (54.5)	
Male	205,060 (45.5)	1814 (49.6)	203,246 (45.5)	
Ethnicity, N (%)				0.007
White	405,819 (90.1)	3316 (90.7)	402,503 (90.1)	
Mixed	17,247 (3.8)	161 (4.4)	17,086 (3.8)	
Asian	16,150 (3.6)	117 (3.2)	16,033 (3.6)	
Black	2789 (0.6)	13 (0.4)	2776 (0.6)	
Chinese	1,524 (0.3)	9 (0.2)	1515 (0.3)	
Other	4383 (1.0)	19 (0.5)	4364 (1.0)	
Missing	2570 (0.6)	19 (0.5)	2551 (0.6)	
Education level, N (%)				<0.001
Degree	145,661 (32.3)	586 (16.0)	145,075 (32.5)	
No degree	295,683 (65.6)	2946 (80.6)	292,737 (65.5)	
Missing	9138 (2.0)	122 (3.3)	9016 (2.0)	
Smoking status, N (%)				<0.001
Never	246,380 (54.7)	499 (13.7)	245,881 (55.0)	
Former	152,785 (33.9)	1595 (43.7)	151,190 (33.8)	
Current	48,660 (10.8)	1518 (41.5)	47,142 (10.6)	
Missing	2657 (0.6)	42 (1.1)	2615 (0.6)	
Smoking duration, mean ± SD	26 ± 13	38 ± 12	26 ± 13	<0.001
Personal history of emphysema/bronchitis, N (%)				<0.001
No	443,241 (98.4)	3324 (91.0)	439,917 (98.5)	
Yes	5823 (1.3)	269 (7.4)	5554 (1.2)	
Missing	1418 (0.3)	61 (1.7)	1357 (0.3)	
Family history of LC, N (%)				<0.001
No	310,558 (68.9)	2107 (57.7)	308,451 (69.0)	
Yes	27,383 (6.1)	402 (11.0)	26,981 (6.0)	
Missing	112,541 (25.0)	1145 (31.3)	111,396 (24.9)	

BMI, body mass index; LC, lung cancer; MHUW, metabolically healthy underweight; MHN, metabolically healthy normal; MHO, metabolically healthy obesity; MHOW, metabolically healthy overweight; MUUW, metabolically unhealthy underweight; MUN, metabolically unhealthy normal; MUO, metabolically unhealthy obesity; MUOW, metabolically unhealthy overweight; SD, standard deviation.

**Table 2:** One-sample MR estimates for the relationship between metabolic obesity phenotypes and incident LC.

Characteristics	No.SNPs ^b^	*F*-Statistics	OR (95%CI)	*p*-Value
Metabolic obesity phenotypes ^a^				
MHUW	12	56	0.98 (0.90, 1.06)	0.55
MUUW	50	463	1.13 (0.95, 1.35)	0.17
MHN			Reference	
MUN	17	281	1.00 (0.98, 1.01)	0.83
MHOW	18	44	1.00 (0.99, 1.01)	0.53
MUOW	51	153	1.00 (1.00, 1.01)	0.24
MHO	112	373	1.01 (1.00, 1.02)	0.07
MUO	207	657	1.00 (0.99, 1.01)	0.86
Trend1	331	637	1.00 (1.00, 1.01)	0.44
Trend2	175	619	1.00 (1.00, 1.00)	0.40
BMI groups				
Underweight	10	31	0.94 (0.85, 1.04)	0.22
Normal			Reference	
Overweight	46	61	1.01 (1.00, 1.02)	0.09
Obesity	466	260	1.01 (0.99, 1.03)	0.30
Trend	490	234	1.01 (0.99, 1.03)	0.29
Metabolic phenotypes				
Healthy			Reference	
Unhealthy	128	295	1.00 (1.00, 1.01)	0.25

^a^ Metabolic obesity phenotypes orders include metabolically healthy underweight (MHUW)/metabolically healthy normal (MHN)/metabolically healthy overweight (MHOW)/metabolically healthy obesity (MHO)/metabolically unhealthy underweight (MUUW)/metabolically unhealthy normal (MUN)/metabolically unhealthy overweight (MUOW)/metabolically unhealthy obesity (MUO) (marked as 1) and MHUW/MUUW/MHN/MUN/MHOW/MUOW/MHO/MUO (marked as 2); ^b^ Number of SNPs after LD control, harmonizing process and removing outliers; we relaxed the instrument p-value threshold (*p* < 5 × 10^6^) for MHUW, MUUW, underweight groups; SNP, single nucleotide polymorphism; OR, odds ratio.

**Table 3:** Two-sample MR estimates for the relationship between metabolic obesity phenotypes and incident LC.

Characteristics	No.SNPs ^b^	OR (95%CI)	*p*-Value	Q Statistic	*p*-Value for Q
Metabolic obesity phenotypes ^a^					
MHUW	10	1.06 (0.93, 1.21)	0.39	22	$6.7\;\times \;10^{-3}$
MUUW	35	0.98 (0.97, 1.00)	0.04	37	0.32
MHN		Reference			
MUN	14	0.97 (0.88, 1.08)	0.60	20	0.10
MHOW	16	1.16 (0.90, 1.48)	0.24	24	0.07
MUOW	46	1.02 (0.93, 1.12)	0.61	56	0.12
MHO	97	1.07 (1.01, 1.14)	0.03	124	0.03
MUO	190	1.11 (1.05, 1.17)	0.001	292	$2.4\;\times \;10^{-6}$
Trend1	294	1.17 (1.07, 1.27)	$2.9\times 10^{-4}$	408	$9.4\;\times \;10^{-6}$
Trend2	154	1.08 (0.99, 1.17)	0.090.089	235	$2.1\;\times \;10^{-5}$
BMI groups					
Underweight	6	1.00 (0.88, 1.13)	0.96	6	0.31
Normal		Reference			
Overweight	38	1.05 (0.84, 1.31)	0.65	67	$2.0\;\times \;10^{-3}$
Obesity	422	1.09 (1.04, 1.14)	0.001	576	$6.8\;\times \;10^{-7}$
Trend	440	1.35 (1.14, 1.60)	$4.9\;\times \;10^{-4}$	611	$1.0\;\times \;10^{-7}$
Metabolic phenotypes					
Healthy		Reference			
Unhealthy	108	1.01 (0.92, 1.11)	0.80	169	$1.2\;\times \;10^{-4}$

^a^ Metabolic obesity phenotypes orders include metabolically healthy underweight (MHUW)/metabolically healthy normal (MHN)/metabolically healthy overweight (MHOW)/metabolically healthy obesity (MHO)/metabolically unhealthy underweight (MUUW)/metabolically unhealthy normal (MUN)/metabolically unhealthy overweight (MUOW)/metabolically unhealthy obesity (MUO) (marked as 1) and MHUW/MUUW/MHN/MUN/MHOW/MUOW/MHO/MUO (marked as 2); ^b^ Number of SNPs after LD control, harmonizing process and removing outliers; SNP, Single nucleotide polymorphism; OR, Odds ratio.

## Data Availability

The UKBB phenotypic data and GWAS data analyzed during the current study are available from the corresponding author on reasonable request.

## Supplementary Materials

The following are available at https://www.mdpi.com/article/10.3390/nu14163370, Figure S1: Flowchart for participant selection, Figure S2: Sex- and age-specific prevalence of metabolic obesity phenotypes at baseline; Figure S3: Stratification analysis by smoking status between metabolic obesity phenotypes and LC risk; Figure S4: Stratification analysis by gender between metabolic obesity phenotypes and LC risk; Figure S5: Stratification analysis by 60 years age between metabolic obesity phenotypes and LC risk; Table S1: Criteria for metabolically healthy; Table S2: Number of LC/non-LC samples after stratified by smoking status; Table S3: Mutually adjusted association of BMI group and metabolic health group with lung cancer; Table S4: Sensitivity analysis for the association between metabolic obesity phenotypes and LC risk; Table S5: Two-sample MR with MR-Egger, weighted median, simple mode and weighted mode methods; Table S6: Multi-stratum one-sample MR estimates for the relationship between metabolic obesity phenotypes and incident LC in never smokers; Table S7: Multi-stratum one-sample MR estimates for the relationship between metabolic obesity phenotypes and incident LC in former smokers; Table S8: Multi-stratum one-sample MR estimates for the relationship between metabolic obesity phenotypes and incident LC in current smokers.

## Abbreviations

The following abbreviations are used in this manuscript:

BMI	Body mass index
BP	Blood pressure
CI	Confidence interval
CRP	C-reactive protein
CSE	Certificate of Secondary Education
DBP	Diastolic pressure
GWAS	Genome-wide association study
HbA1c	Hemoglobin A1c
HDL-C	High-density lipoprotein cholesterol
HES	Hospital episodes statistics
HR	Hazard ratio
hs-CRP	High-sensitivity C-reactive protein
ICD	International Classification of Diseases
IV	Instrumental variable
LC	Lung cancer
LDL-C	Low-density lipoprotein cholesterol
MH	Metabolically health
MHN	Metabolically healthy normal
MHO	Metabolically healthy obesity
MHOW	Metabolically healthy overweight
MR	Mendelian randomization
MUN	Metabolically unhealthy normal
MUO	Metabolically unhealthy obesity
MUOW	Metabolically unhealthy overweight
NVQ	National Vocational Qualification
OR	Odds ratio
SBP	Systolic pressure
SD	Standard deviation
SNP	Single nucleotide polymorphism
TAG	Triacylglycerols
2SLS	Two-stage least-squares regression
