# Supplementary material for: Metabolic Obesity Phenotypes and Risk of Lung Cancer: A Prospective Cohort Study of 450,482 UK Biobank Participants
Source: Nutrients. 2022 Aug 17;14(16):3370. doi: 10.3390/nu14163370 (PMC9414360; doi:10.3390/nu14163370)
Supplement: Supplementary file 1 [file nutrients-14-03370-s001.zip › nutrients-1844238-supplementary.pdf]

Supplementary to manuscript:

Metabolic obesity phenotypes and risk of lung cancer: a prospective cohort study of 450,482 UK Biobank participants

Supplementary

Supplementary Table S1. Criteria for metabolically healthy

Supplementary Table S2. Number of LC/non-LC samples after stratified by smoking status

Supplementary Table S3. Mutually adjusted association of BMI group and metabolic health group with lung cancer

Supplementary Table S4. Sensitivity analysis for the association between metabolic obesity phenotypes and LC risk

Supplementary Table S5. Two-sample MR with MR-Egger, weighted median, simple mode and weighted mode methods

Supplementary Table S6. Multi-stratum one-sample MR estimates for the relationship between metabolic obesity phenotypes and incident LC in never smokers

Supplementary Table S7. Multi-stratum one-sample MR estimates for the relationship between metabolic obesity phenotypes and incident LC in former smokers

Supplementary Table S8. Multi-stratum one-sample MR estimates for the relationship between metabolic obesity phenotypes and incident LC in current smokers

Supplementary Figure S1. Flowchart for participant selection

Supplementary Figure S2. Sex- and age-specific prevalence of metabolic obesity

phenotypes at baseline

Supplementary Figure S3. Stratification analysis by smoking status between metabolic obesity phenotypes and LC risk

Supplementary Figure S4. Stratification analysis by gender between metabolic obesity phenotypes and LC risk

Supplementary Figure S5. Stratification analysis by 60 years age between metabolic obesity phenotypes and LC risk

Supplementary Table S1. Criteria for metabolically healthy

Biomarker	Criteria for Metabolically Healthy
BP	SBP <130 mmHg and DBP <80 mmHg and no antihypertensive medications
CRP	CRP <3 mg/L
Triacylglycerols	Triacylglycerols <2.3 mmol/L
LDL-C	LDL-C<3 mmol/L and no cholesterol lowering medications
HDL-C	HDL-C>1 mmol/L
Glycated hemoglobin	HbA1c <42 mmol/mol (6%) and no diabetes medications

BP, Blood pressure; SBP, Systolic pressure; DBP, Diastolic pressure; CRP, C-reactive protein; LDL-C, Low-density lipoprotein cholesterol; HDL-C, High-density lipoprotein cholesterol.

Supplementary Table S2. Number of LC/non-LC samples after stratified by smoking status

	Never smokers		Former smokers		Current smokers	
	LC (N = 499)	Non-LC (N = 245,881)	LC (N = 1,595)	Non-LC (N = 151,190)	LC (N = 1,518)	Non-LC (N = 47,142)
Metabolic obesity phenotypes						
MHUW	60	27,013	307	20,059	286	7,388
MUUW	0	32	3	10	6	32
MHN	115	60,282	198	28,969	301	10,145
MUN	43	18,414	110	12,583	71	2,697
MHOW	107	55,233	313	34,087	172	8,416
MUOW	0	1,015	3	355	21	367
MHO	25	8,649	66	4,917	176	3,040
MUO	54	26,929	300	21,368	183	5,876
BMI groups						
Underweight	1	1,322	10	453	32	503
Normal	173	85,548	335	41,784	596	16,244
Overweight	200	101,440	746	66,368	556	19,373
Obesity	122	56,264	494	41,880	311	10,612
Metabolic phenotypes						
Healthy	266	135,364	626	76,188	569	21,717
Unhealthy	140	62,945	678	46,554	662	16,431

Supplementary Table S3. Mutually adjusted association of BMI groups and metabolic health groups with lung cancer

Variable	LC/Non-LC	HR (95% CI) [a]	*P* value
BMI categories			
Underweight	44 / 2,288	1.42 (0.87, 2.32)	0.16
Normal	1,113 / 144,124	Reference	
Overweight	1,514 / 188,144	0.87 (0.77, 0.99)	0.04
Obesity	945 / 109,463	0.81 (0.70, 0.94)	0.005
Metabolic health status			
Healthy	1,474 / 234,326	Reference	
Unhealthy	1,495 / 126,672	1.17 (1.05, 1.31)	0.004

[a]The Cox proportional hazards regression model was adjusted for age, sex, education level, smoking status, family history of LC and personal history of emphysema/bronchitis.

Supplementary Table S4. Sensitivity analysis for the association between metabolic obesity phenotypes and LC risk

Model	Evaluation method	Characteristics	HR (95%CI)	*P*-value
1	Inclusion of incidence lung cancer to the first 1 year of follow-up	Metabolic obesity phenotypes		
		MHUW	1.12 (0.63,2.00)	0.70
		MUUW	3.17 (1.30,7.68)	0.01
		MHN	Reference	
		MUN	1.06 (0.87,1.30)	0.57
		MHOW	0.82 (0.70,0.96)	0.01
		MUOW	1.01 (0.86,1.17)	0.94
		MHO	0.75 (0.60,0.94)	0.01
		MUO	0.92 (0.78,1.08)	0.29
		Trend	0.98 (0.95,1.00)	0.10
		BMI groups		
		Underweight	1.37 (0.89,2.13)	0.16
		Normal	Reference	
		Overweight	0.89 (0.79,0.99)	0.03
		Obesity	0.83 (0.73,0.94)	0.004
		Trend	0.90 (0.85,0.96)	0.001
		Metabolic phenotypes		
		Healthy	Reference	
		Unhealthy	1.12 (1.01,1.24)	0.03
2	Additional adjustment for family history of any cancer and drinking status	Metabolic obesity phenotypes		
		MHUW	1.04 (0.49,2.22)	0.92
		MUUW	1.32 (0.18,9.49)	0.78
		MHN	Reference	
		MUN	1.00 (0.76,1.31)	0.98
		MHOW	0.88 (0.72,1.08)	0.24
		MUOW	0.96 (0.78,1.18)	0.70
		MHO	0.69 (0.51,0.93)	0.02
		MUO	0.87 (0.71,1.08)	0.21
		Trend	0.97 (0.93,1.00)	0.08
		BMI groups		
		Underweight	0.97 (0.50,1.88)	0.92
		Normal	Reference	
		Overweight	0.88 (0.76,1.01)	0.08
		Obesity	0.81 (0.69,0.95)	0.01
		Trend	0.90 (0.83,0.98)	0.01
		Metabolic phenotypes		
		Healthy	Reference	
		Unhealthy	1.05 (0.92,1.20)	0.47
3	Additional adjustment for age at first smoking, number of cigarettes smoked daily, package	Metabolic obesity phenotypes		
		MHUW	1.47 (0.47,4.64)	0.51
		MUUW	11.49 (2.81,46.96)	0.001
		MHN	Reference	
		MUN	0.98 (0.67,1.43)	0.90

	year, time since last smoking (in former smokers)	MHOW	1.00 (0.79,1.26)	0.98
		MUOW	1.10 (0.86,1.40)	0.46
		MHO	0.68 (0.49,0.94)	0.02
		MUO	0.93 (0.73,1.19)	0.58
		Trend	0.97 (0.93,1.01)	0.14
		BMI groups		
		Underweight	2.35 (1.04,5.32)	0.04
		Normal	Reference	
		Overweight	1.02 (0.85,1.21)	0.87
		Obesity	0.82 (0.68,0.99)	0.04
		Trend	0.88 (0.81,0.97)	0.008
		Metabolic phenotypes		
		Healthy	Reference	
		Unhealthy	1.09 (0.94,1.26)	0.26
4	Exclusion of participants that were former smokers	Metabolic obesity phenotypes		
		MHUW	1.03 (0.52,2.01)	0.94
		MUUW	2.12 (0.68,6.67)	0.20
		MHN	Reference	
		MUN	1.01 (0.79,1.30)	0.93
		MHOW	0.69 (0.55,0.87)	0.002
		MUOW	0.90 (0.73,1.11)	0.31
		MHO	0.92 (0.66,1.28)	0.62
		MUO	0.81 (0.64,1.03)	0.09
		Trend	0.96 (0.92,1.00)	0.06
		BMI groups		
		Underweight	1.11 (0.65,1.91)	0.70
		Normal	Reference	
		Overweight	0.79 (0.68,0.92)	0.002
		Obesity	0.82 (0.68,0.98)	0.03
		Trend	0.89 (0.81,0.97)	0.006
		Metabolic phenotypes		
		Healthy	Reference	
		Unhealthy	1.04 (0.90,1.20)	0.61
5	Exclusion of participants that were non-Europeans	Metabolic obesity phenotypes		
		MHUW	1.03 (0.55,1.94)	0.93
		MUUW	2.90 (1.08,7.81)	0.04
		MHN	Reference	
		MUN	1.07 (0.87,1.33)	0.52
		MHOW	0.84 (0.71,1.00)	0.05
		MUOW	1.02 (0.87,1.21)	0.77
		MHO	0.81 (0.64,1.02)	0.07
		MUO	0.96 (0.81,1.14)	0.62
		Trend	0.99 (0.96,1.02)	0.38
		BMI groups		
		Underweight	1.24 (0.76,2.01)	0.39
		Normal	Reference	

		Overweight	0.92 (0.81,1.03)	0.14
		Obesity	0.88 (0.78,1.01)	0.07
		Trend	0.94 (0.88,1.00)	0.04
		Metabolic phenotypes		
		Healthy	Reference	
		Unhealthy	1.12 (1.01,1.25)	0.03
6	Exclusion of participants with missing covariates	Metabolic obesity phenotypes		
		MHUW	1.14 (0.64,2.03)	0.66
		MUUW	3.24 (1.33,7.87)	0.009
		MHN	Reference	
		MUN	1.04 (0.85,1.28)	0.72
		MHOW	0.82 (0.70,0.96)	0.02
		MUOW	1.00 (0.86,1.17)	0.97
		MHO	0.76 (0.61,0.95)	0.02
		MUO	0.92 (0.78,1.08)	0.33
		Trend	0.98 (0.95,1.01)	0.13
		BMI groups		
		Underweight	1.40 (0.90,2.17)	0.13
		Normal	Reference	
		Overweight	0.89 (0.80,0.99)	0.04
		Obesity	0.84 (0.74,0.96)	0.009
		Trend	0.91 (0.86,0.97)	0.003
		Metabolic phenotypes		
		Healthy	Reference	
		Unhealthy	1.11 (1.00,1.23)	0.04
7	Additional adjustment for dietary patterns (red and processed meat intake, fruit and vegetable intake) and physical activity (IPAQ)	Metabolic obesity phenotypes		
		MHUW	0.93 (0.44,1.98)	0.85
		MUUW	3.07 (0.98,9.65)	0.06
		MHN	Reference	
		MUN	1.08 (0.86,1.36)	0.51
		MHOW	0.81 (0.67,0.97)	0.03
		MUOW	1.01 (0.85,1.21)	0.90
		MHO	0.77 (0.59,1.00)	0.047
		MUO	0.88 (0.72,1.06)	0.18
		Trend1	0.97 (0.94,1.01)	0.10
		Trend2	1.01 (0.98,1.04)	0.48
		BMI groups		
		Underweight	1.25 (0.72,2.18)	0.43
		Normal	Reference	
		Overweight	0.89 (0.78,1.01)	0.07
		Obesity	0.81 (0.70,0.94)	0.005
		Trend	0.90 (0.83,0.96)	0.003
		Metabolic phenotypes		
		Healthy	Reference	
		Unhealthy	1.11 (0.99,1.25)	0.08
8	Additional	Metabolic obesity phenotypes		

adjustment for waist hip ratio (continuous)	MHUW	1.23 (0.69,2.19)	0.49
	MUUW	3.48 (1.43,8.46)	0.006
	MHN	Reference	
	MUN	1.00 (0.81,1.23)	0.98
	MHOW	0.77 (0.65,0.91)	0.002
	MUOW	0.90 (0.77,1.07)	0.23
	MHO	0.67 (0.53,0.85)	0.001
	MUO	0.79 (0.65,0.95)	0.01
	Trend1	0.95 (0.91,0.98)	0.002
	Trend2	1.00 (0.98,1.02)	0.98
	BMI groups		
	Underweight	1.54 (0.99,2.39)	0.05
	Normal	Reference	
	Overweight	0.82 (0.73,0.92)	0.001
	Obesity	0.72 (0.63,0.84)	9.7×10^{-6}
	Trend	0.84 (0.79,0.90)	2.0×10^{-6}
	Metabolic phenotypes		
	Healthy	Reference	
	Unhealthy	1.07 (0.96,1.20)	0.19

Supplementary Table S5. Two-sample MR with MR-Egger, weighted median, simple mode and weighted mode methods

	Method	No.SNPs	OR (95%CI)	P-value
Metabolic obesity phenotypes				
MHUW	MR Egger	10	0.90 (0.67,1.23)	0.54
MHUW	Weighted median	10	1.12 (0.99,1.27)	0.08
MHUW	Simple mode	10	1.17 (0.95,1.45)	0.17
MHUW	Weighted mode	10	1.13 (0.94,1.35)	0.22
MUUW	MR Egger	35	0.96 (0.88,1.04)	0.30
MUUW	Weighted median	35	0.99 (0.97,1.01)	0.50
MUUW	Simple mode	35	1.00 (0.96,1.05)	0.97
MUUW	Weighted mode	35	1.00 (0.96,1.04)	0.97
MHN			Reference	
MUN	MR Egger	14	1.12 (0.87,1.43)	0.41
MUN	Weighted median	14	1.02 (0.91,1.15)	0.75
MUN	Simple mode	14	1.00 (0.83,1.22)	0.96
MUN	Weighted mode	14	1.05 (0.93,1.18)	0.42
MHOW	MR Egger	16	1.39 (0.48,4.01)	0.55
MHOW	Weighted median	16	1.23 (0.92,1.64)	0.16
MHOW	Simple mode	16	1.64 (0.91,2.96)	0.12
MHOW	Weighted mode	16	1.35 (0.84,2.18)	0.24
MUOW	MR Egger	46	1.11 (0.90,1.38)	0.33
MUOW	Weighted median	46	1.02 (0.90,1.15)	0.81
MUOW	Simple mode	46	1.07 (0.84,1.38)	0.57
MUOW	Weighted mode	46	1.06 (0.92,1.24)	0.43
MHO	MR Egger	97	1.03 (0.85,1.25)	0.76
MHO	Weighted median	97	1.04 (0.95,1.14)	0.36
MHO	Simple mode	97	1.04 (0.84,1.29)	0.74
MHO	Weighted mode	97	1.02 (0.87,1.18)	0.84
MUO	MR Egger	190	1.01 (0.85,1.21)	0.90
MUO	Weighted median	190	1.09 (1.01,1.18)	0.02
MUO	Simple mode	190	1.06 (0.85,1.33)	0.60
MUO	Weighted mode	190	1.05 (0.89,1.23)	0.57
MHO Trend1	MR Egger	294	1.01 (0.79,1.31)	0.91
MHO Trend1	Weighted median	294	1.16 (1.03,1.31)	0.02
MHO Trend1	Simple mode	294	1.09 (0.73,1.62)	0.68
MHO Trend1	Weighted mode	294	1.06 (0.82,1.37)	0.65
MHO Trend2	MR Egger	154	0.99 (0.82,1.19)	0.89
MHO Trend2	Weighted median	154	1.08 (0.97,1.22)	0.16
MHO Trend2	Simple mode	154	1.13 (0.86,1.48)	0.39
MHO Trend2	Weighted mode	154	1.08 (0.94,1.24)	0.28
BMI groups				
Underweight	MR Egger	6	1.05 (0.81,1.36)	0.74
Underweight	Weighted median	6	0.95 (0.81,1.11)	0.49
Underweight	Simple mode	6	0.92 (0.73,1.16)	0.49
Underweight	Weighted mode	6	0.91 (0.73,1.14)	0.45
Normal			Reference	
Overweight	MR Egger	38	0.95 (0.46,1.94)	0.88

Overweight	Weighted median	38	1.04 (0.81,1.34)	0.76
Overweight	Simple mode	38	1.27 (0.75,2.15)	0.38
Overweight	Weighted mode	38	1.02 (0.68,1.53)	0.92
Obesity	MR Egger	422	1.00 (0.87,1.16)	0.98
Obesity	Weighted median	422	1.07 (1.00,1.15)	0.05
Obesity	Simple mode	422	1.03 (0.81,1.30)	0.81
Obesity	Weighted mode	422	1.03 (0.89,1.19)	0.70
BMI Trend	MR Egger	440	1.02 (0.61,1.70)	0.95
BMI Trend	Weighted median	440	1.21 (0.95,1.53)	0.12
BMI Trend	Simple mode	440	0.92 (0.40,2.12)	0.84
BMI Trend	Weighted mode	440	0.98 (0.56,1.70)	0.93
Metabolic phenotypes				
Healthy			Reference	
Unhealthy	MR Egger	108	1.00 (0.85,1.18)	0.99
Unhealthy	Weighted median	108	1.03 (0.91,1.16)	0.61
Unhealthy	Simple mode	108	1.15 (0.89,1.48)	0.30
Unhealthy	Weighted mode	108	1.07 (0.96,1.20)	0.23

Supplementary Table S6. Multi-stratum one-sample MR estimates for the relationship between metabolic obesity phenotypes and incident LC in never smokers

	No.SNPs[b]	F-statistics	OR (95%CI)	P-value
Metabolic obesity phenotypes[a]				
MHUW	15	133	1.02 (0.98,1.06)	0.40
MUUW	67	710	1.05 (0.93,1.18)	0.46
MHN			Reference	
MUN	13	275	1.00 (0.99,1.01)	0.57
MHOW	6	53	0.99 (0.98,1.00)	0.07
MUOW	19	320	1.00 (0.99,1.00)	0.24
MHO	39	465	1.00 (0.99,1.00)	0.35
MUO	63	686	1.00 (1.00,1.01)	0.46
Trend1	126	624	1.00 (1.00,1.00)	0.14
Trend2	51	358	1.00 (1.00,1.00)	0.48
BMI groups				
Underweight	17	98	1.02 (0.98,1.06)	0.36
Normal			Reference	
Overweight	11	45	1.00 (0.99,1.00)	0.35
Obesity	163	571	1.00 (1.00,1.00)	0.89
Trend	178	691	1.00 (1.00,1.00)	0.45
Metabolic phenotypes				
Healthy			Reference	
Unhealthy	46	418	1.00 (1.00,1.00)	0.45

[a]Metabolic obesity phenotypes orders include metabolically healthy normal (MHN)/metabolically healthy overweight (MHOW)/metabolically healthy obesity (MHO)/metabolically unhealthy normal (MUN)/metabolically unhealthy overweight (MUOW)/metabolically unhealthy obesity (MUO) (marked as 1) and MHN/MUN/MHOW/MUOW/MHO/MUO (marked as 2);

[b]Number of SNPs after LD control, harmonizing process and removing outliers; we relaxed the instrument *P*-value threshold ($P < 5 \times 10^{-6}$) for MHUW, MUUW, underweight groups;

SNP, Single nucleotide polymorphism; OR, Odds ratio.

Supplementary Table S7. Multi-stratum one-sample MR estimates for the relationship between metabolic obesity phenotypes and incident LC in former smokers

	No.SNPs[b]	F-statistics	OR (95%CI)	P-value
Metabolic obesity phenotypes[a]				
MHUW	25	209	1.01 (0.94,1.07)	0.84
MUUW	78	1169	0.99 (0.83,1.18)	0.89
MHN			Reference	
MUN	16	129	1.01 (0.98,1.03)	0.47
MHOW	21	74	0.99 (0.98,1.01)	0.55
MUOW	8	83	0.99 (0.97,1.01)	0.43
MHO	16	172	1.00 (0.99,1.02)	0.49
MUO	28	289	1.00 (0.99,1.01)	0.98
Trend1	46	154	1.00 (1.00,1.00)	0.78
Trend2	20	106	1.00 (1.00,1.00)	0.99
BMI groups				
Underweight	20	98	1.00 (0.91,1.09)	0.98
Normal			Reference	
Overweight	37	70	1.01 (0.99,1.02)	0.50
Obesity	68	381	1.00 (0.99,1.01)	0.89
Trend	74	240	1.00 (0.99,1.00)	0.70
Metabolic phenotypes				
Healthy			Reference	
Unhealthy	22	132	1.00 (0.99,1.01)	0.79

[a]Metabolic obesity phenotypes orders include metabolically healthy normal (MHN)/metabolically healthy overweight (MHOW)/metabolically healthy obesity (MHO)/metabolically unhealthy normal (MUN)/metabolically unhealthy overweight (MUOW)/metabolically unhealthy obesity (MUO) (marked as 1) and MHN/MUN/MHOW/MUOW/MHO/MUO (marked as 2);

[b]Number of SNPs after LD control, harmonizing process and removing outliers; we relaxed the instrument *P*-value threshold ($P < 5 \times 10^{-6}$) for MHUW, MUUW, MUN, MHOW, underweight, overweight groups;

SNP, Single nucleotide polymorphism; OR, Odds ratio.

Supplementary Table S8. Multi-stratum one-sample MR estimates for the relationship between metabolic obesity phenotypes and incident LC in current smokers

	No.SNPs[b]	F-statistics	OR (95%CI)	P-value
Metabolic obesity phenotypes[a]				
MHUW	16	337	1.06 (0.96,1.18)	0.22
MUUW	69	1179	1.14 (0.95,1.38)	0.16
MHN			Reference	
MUN	10	190	1.03 (0.97,1.09)	0.34
MHOW	20	259	0.98 (0.95,1.01)	0.28
MUOW	16	213	0.99 (0.95,1.03)	0.77
MHO	7	145	1.00 (0.94,1.07)	0.89
MUO	5	143	0.97 (0.92,1.02)	0.21
Trend1	6	84	1.00 (0.99,1.01)	0.46
Trend2	8	105	0.99 (0.98,1.00)	0.04
BMI groups				
Underweight	18	281	1.06 (0.94,1.18)	0.35
Normal			Reference	
Overweight	19	124	1.01 (0.98,1.05)	0.54
Obesity	10	182	0.98 (0.94,1.01)	0.17
Trend	7	114	0.97 (0.95,0.99)	0.007
Metabolic phenotypes				
Healthy			Reference	
Unhealthy	9	128	0.97 (0.94,1.01)	0.12

[a]Metabolic obesity phenotypes orders include metabolically healthy normal (MHN)/metabolically healthy overweight (MHOW)/metabolically healthy obesity (MHO)/metabolically unhealthy normal (MUN)/metabolically unhealthy overweight (MUOW)/metabolically unhealthy obesity (MUO) (marked as 1) and MHN/MUN/MHOW/MUOW/MHO/MUO (marked as 2);

[b]Number of SNPs after LD control, harmonizing process and removing outliers; we relaxed the instrument *P*-value threshold ($P < 5 \times 10^{-6}$) for MHUW, MUUW, MUN, MHOW, underweight, overweight groups;

SNP, Single nucleotide polymorphism; OR, Odds ratio.

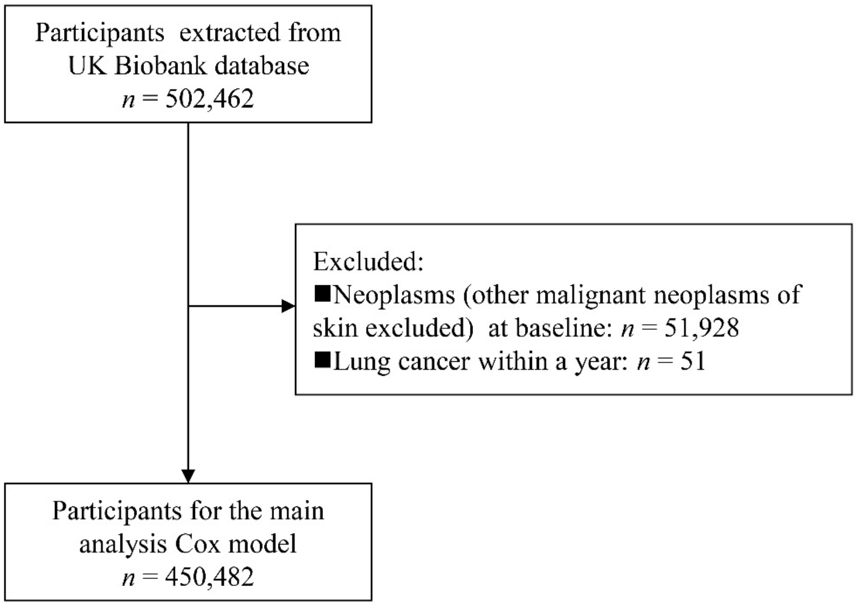

Supplementary Figure S1. Flowchart for participant selection

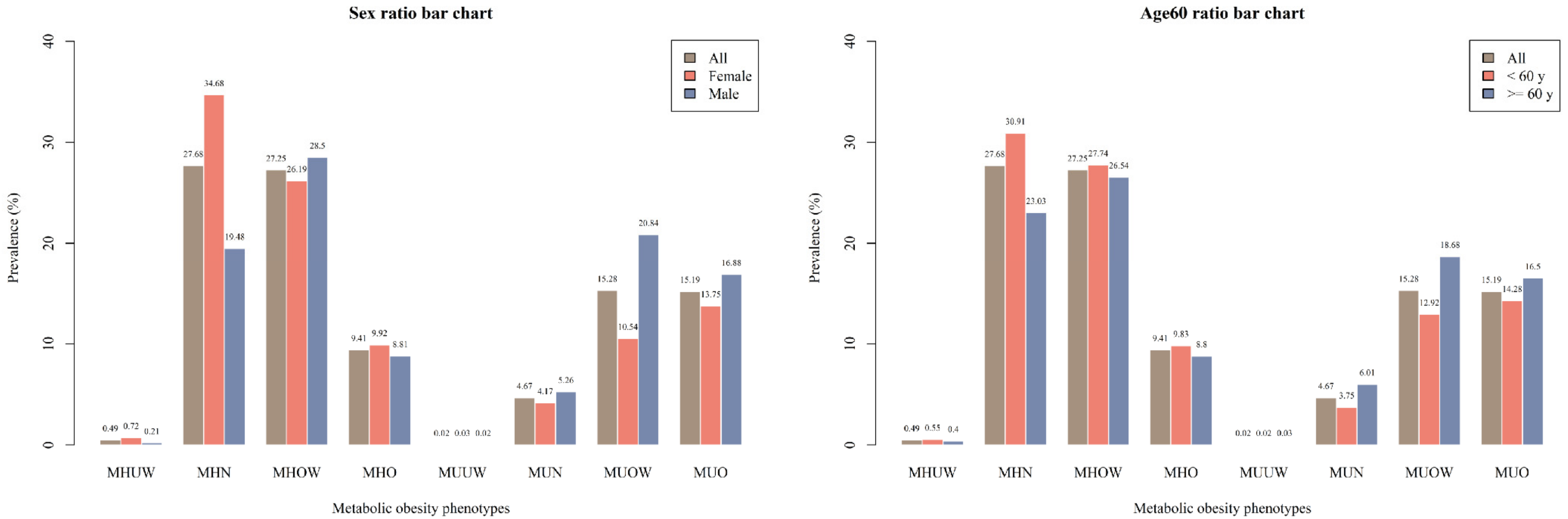

Supplementary Figure S2. Sex- and age-specific prevalence of metabolic obesity phenotypes at baseline

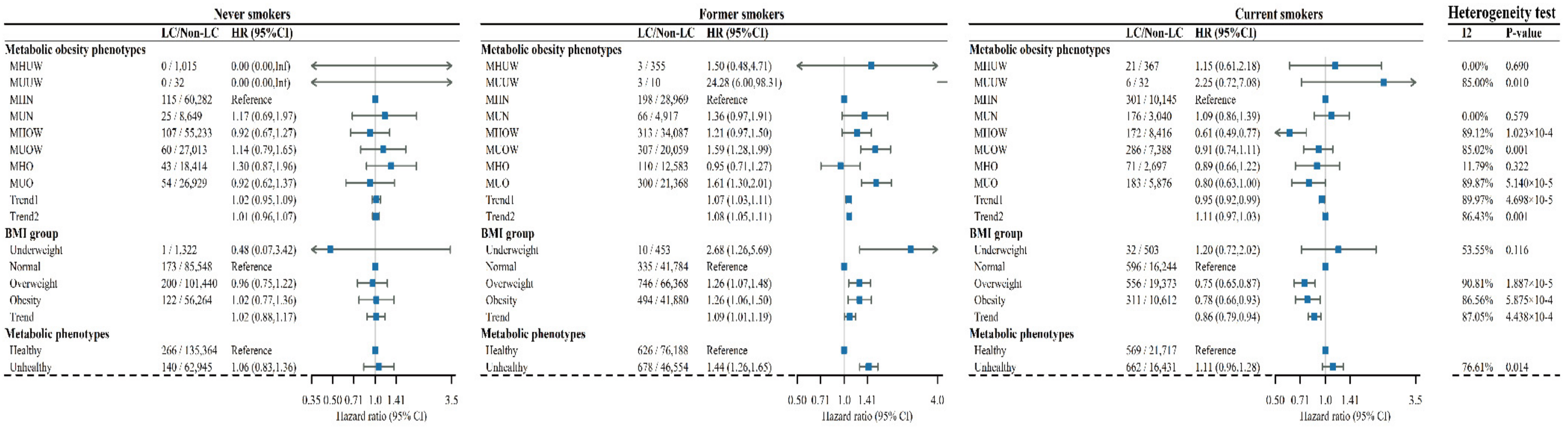

Supplementary Figure S3. Stratification analysis by smoking status between metabolic obesity phenotypes and LC risk

HRs: hazard ratios; LC: lung cancer; BMI, body mass index; MHUW, metabolically healthy underweight; MHN, metabolically healthy normal; MHO, metabolically healthy obesity; MHOW, metabolically healthy overweight; MUUW, metabolically unhealthy underweight; MUN, metabolically unhealthy normal; MUO, metabolically unhealthy obesity; MUOW, metabolically unhealthy overweight.

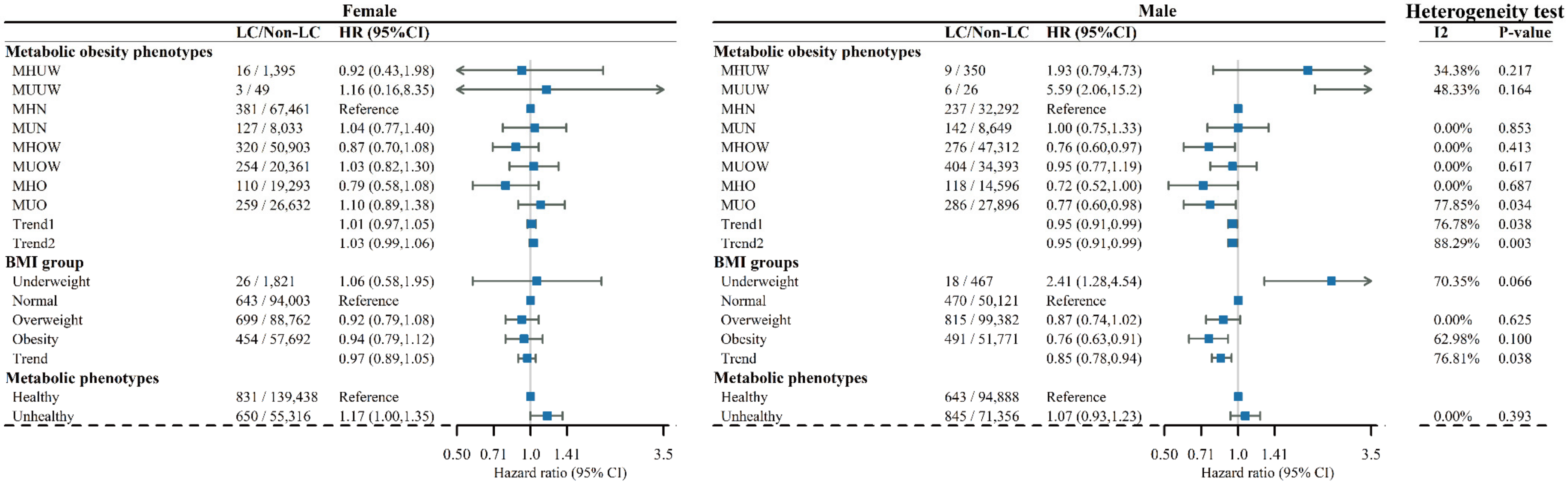

Female

	LC/Non-LC	HR (95%CI)
**Metabolic obesity phenotypes**		
MHUW	16 / 1,395	0.92 (0.43,1.98)
MUUW	3 / 49	1.16 (0.16,8.35)
MHN	381 / 67,461	Reference
MUN	127 / 8,033	1.04 (0.77,1.40)
MHOW	320 / 50,903	0.87 (0.70,1.08)
MUOW	254 / 20,361	1.03 (0.82,1.30)
MHO	110 / 19,293	0.79 (0.58,1.08)
MUO	259 / 26,632	1.10 (0.89,1.38)
Trend1		1.01 (0.97,1.05)
Trend2		1.03 (0.99,1.06)
**BMI group**		
Underweight	26 / 1,821	1.06 (0.58,1.95)
Normal	643 / 94,003	Reference
Overweight	699 / 88,762	0.92 (0.79,1.08)
Obesity	454 / 57,692	0.94 (0.79,1.12)
Trend		0.97 (0.89,1.05)
**Metabolic phenotypes**		
Healthy	831 / 139,438	Reference
Unhealthy	650 / 55,316	1.17 (1.00,1.35)

Male / **Heterogeneity test**

	LC/Non-LC	HR (95%CI)	I2	P-value
**Metabolic obesity phenotypes**				
MHUW	9 / 350	1.93 (0.79,4.73)	34.38%	0.217
MUUW	6 / 26	5.59 (2.06,15.2)	48.33%	0.164
MHN	237 / 32,292	Reference		
MUN	142 / 8,649	1.00 (0.75,1.33)	0.00%	0.853
MHOW	276 / 47,312	0.76 (0.60,0.97)	0.00%	0.413
MUOW	404 / 34,393	0.95 (0.77,1.19)	0.00%	0.617
MHO	118 / 14,596	0.72 (0.52,1.00)	0.00%	0.687
MUO	286 / 27,896	0.77 (0.60,0.98)	77.85%	0.034
Trend1		0.95 (0.91,0.99)	76.78%	0.038
Trend2		0.95 (0.91,0.99)	88.29%	0.003
**BMI groups**				
Underweight	18 / 467	2.41 (1.28,4.54)	70.35%	0.066
Normal	470 / 50,121	Reference		
Overweight	815 / 99,382	0.87 (0.74,1.02)	0.00%	0.625
Obesity	491 / 51,771	0.76 (0.63,0.91)	62.98%	0.100
Trend		0.85 (0.78,0.94)	76.81%	0.038
**Metabolic phenotypes**				
Healthy	643 / 94,888	Reference		
Unhealthy	845 / 71,356	1.07 (0.93,1.23)	0.00%	0.393

Supplementary Figure S4. Stratification analysis by gender between metabolic obesity phenotypes and LC risk

HRs: hazard ratios; LC: lung cancer; BMI, body mass index; MHUW, metabolically healthy underweight; MHN, metabolically healthy normal; MHO, metabolically healthy obesity; MHOW, metabolically healthy overweight; MUUW, metabolically unhealthy underweight; MUN, metabolically unhealthy normal; MUO, metabolically unhealthy obesity; MUOW, metabolically unhealthy overweight.

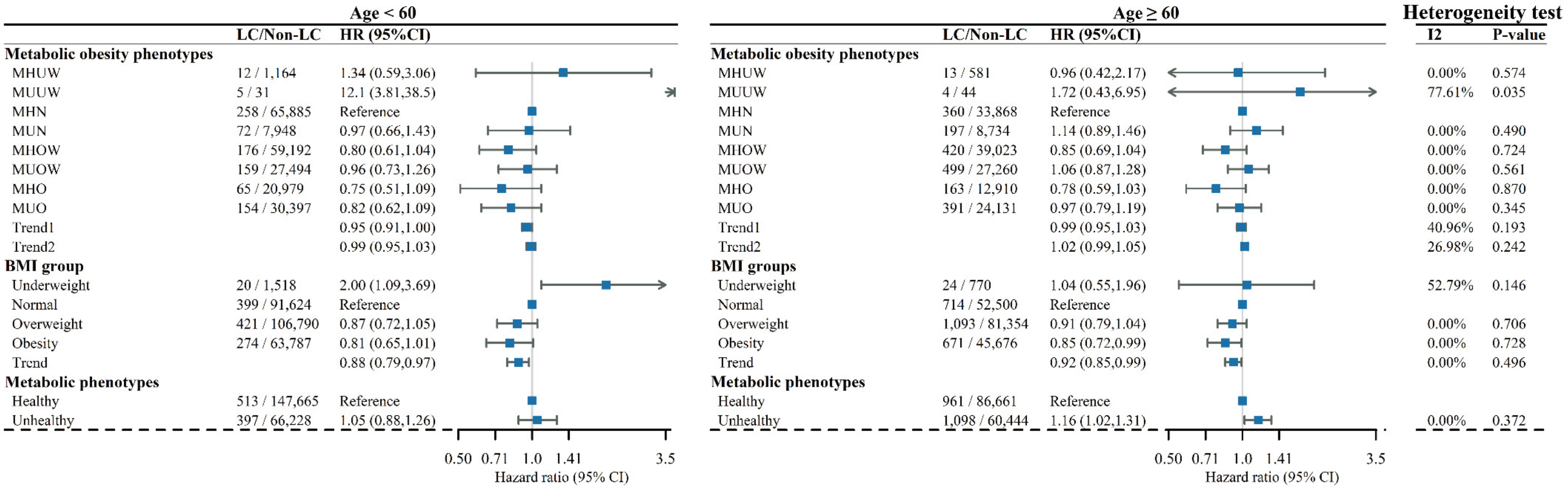

Supplementary Figure S5. Stratification analysis by 60 years age between metabolic obesity phenotypes and LC risk

HRs: hazard ratios; LC: lung cancer; BMI, body mass index; MHUW, metabolically healthy underweight; MHN, metabolically healthy normal; MHO, metabolically healthy obesity; MHOW, metabolically healthy overweight; MUUW, metabolically unhealthy underweight; MUN, metabolically unhealthy normal; MUO, metabolically unhealthy obesity; MUOW, metabolically unhealthy overweight.
